# Editorial: Cross-disciplinary approaches to characterize gait and posture disturbances in aging and related diseases, Volume II

**DOI:** 10.3389/fbioe.2024.1422815

**Published:** 2024-05-30

**Authors:** Simone Tassani, Claudio Belvedere, Juan Ramírez, Giorgio Davico

**Affiliations:** ^1^ Department of Engineering, Universitat Pompeu Fabra, Barcelona, Spain; ^2^ Movement Analysis Laboratory, IRCCS Istituto Ortopedico Rizzoli, Bologna, Italy; ^3^ National University of Colombia, Medellin, Colombia; ^4^ Alma Mater Studiorum, University of Bologna, Bologna, Italy

**Keywords:** gait, posture, multi-factorial analysis, cross-disciplinary, repeatability, aging

Cross-disciplinary research is the space where different disciplines meet and generate new knowledge. However, publishing multidisciplinary research can be difficult since the advances presented in the single disciplines might be small or negligible if considered independently, and the organization of the work can be extremely challenging, starting from the development of a common language and knowledge base and dealing proactively with misunderstandings (Domino et al., 2007; Daniel et al., 2022). Nonetheless, the union of the different areas can be knowledge itself (Priaulx and Weinel, 2018). For these reasons, this Research Topic: “*Cross-Disciplinary Approaches to Characterize Gait and Posture Disturbances in Aging and Related Diseases, Volume II*” gives once again space to multidisciplinary research in gait and posture to help increase the body of evidence at the intersection between the various scientific disciplines and research fields that focus and/or impact on gait and posture.

Great global challenges require cross-disciplinary spaces where science, humanities, and culture can meet and develop solutions for great global problems. For instance, the planetary wellbeing initiative (Antó et al., 2021; Kortetmäki et al., 2021) is considering the loop of changes made by humans to our lifestyle, which in turn affect human health. Situations of high emotional distress, created by the society we have developed, have been seen to promote musculoskeletal disorders, already at a young age (Diepenmaat et al., 2006). In this framework Tassani et al. evaluated the relationship among posture, breathing, and anxiety, therefore exploring the interaction between physical and emotional spheres in University students. Similarly, Kong et al. explored the influence of cognitive load on the trajectory of the center of pressure during gait initiation in young males with excess weight. Cognitive and emotional burdens are often ignored in the study of gait and posture until they reach major relevance (Canales et al., 2017) when any approach to revert the dynamic of the pathology is likely very difficult. For this reason, working on prevention is paramount and even if the focus of the Research Topic is on aging-related diseases, we found it relevant to give space to studies performed on healthy subjects (De Blasiis et al., Peiffer et al.), or where the aging process was simulated in young volunteers (Ma et al.). The mentioned studies are all presenting balances and stability assessments related to fall prediction and fall-related injuries, which again, for a long time was mainly related to the elderly, but is becoming a common problem also in the younger population. It is also very important to develop studies on children (Jiang et al.) and on how posture variations can affect sensitive subjects during development, with changes that can disturb their adulthood.

The first volume of this Research Topic gave space to age-related functionality reduction and the combination of imaging techniques for the study of gait. The relation between gait and imaging technique is probably one of the most common cross-disciplinary approaches in motion analysis (Bohnen and Jahn, 2013). These Research Topic are covered in the work by Ricciardi et al. The study presents how the influence of soft-tissue composition on gait can be evaluated on a large cohort using imaging techniques.

Osteoarthritis is also a common aging disorder, that has negative consequences as disability and societal costs (Hunter and Bierma-Zeinstra, 2019). In the optics of prevention, kinetic and kinematic studies have been developed (Stief et al., 2018). Drongelen et al. have studied the effect of Total Hip Replacement, an end-stage treatment for this pathology, over a bipedal stance. Even if the therapy was found to have a positive effect on the standing position and load distribution of the patients, preventive therapy directed at the disproportionate load distribution might have reduced the progression of the disease.

Another typical problem in multifactorial and multivariate motion studies is the large number of features to be considered in the analysis (Benedetti et al., 1998). For this reason, dimensionality reduction and feature selection of the most appropriate and representative features is an important matter that again finds space in this Research Topic (Ulrich et al., Sato et al.). For example, when evaluating the construct validity of the trunk impairment scale in patients with serious physical problems, like stroke and Parkinson’s disease, correlations and multiple regression analysis showed that this scale may evaluate the trunk function related to balance function, disease severity, activities of daily living function, lower limb strength, but the relevant factors were constructed with three different aspects, and this differs from other factors that included many other aspects. Similarly, in the evaluation of medial knee osteoarthritis, several parameters related to knee moments are considered, which differ with the severity of osteoarthritis, but if properly combined can provide the possibility of developing a severity index. Considering a larger number of parameters helps improve the description of a specific issue, but in turn, is likely to increase the overall complexity of the study design. The possibility to reduce and combine parameters is often desirable and therefore does not surprise that advanced multivariate analysis and machine learning techniques are more and more integrated into movement analysis (Phinyomark et al., 2018) making feature reduction approaches another discipline that nowadays crosses gait biomechanics.

Through this Research Topic and the related articles collected therein, we hope to offer the reader new insights into a more comprehensive approach to the study of human motion and its use in research as well as in clinics, again addressing the interaction of gait with cognitive conditions and the integration of multiple techniques for gait analysis. Working with multivariate approach and considering high dimensionality is becoming mandatory for a holistic vision of gait analysis. Nonetheless, with this Research Topic we wanted to give more space especially to the effect that cognitive and emotional loads can have over gait and posture. Unfortunately, the study of the disease risks to shadow the understanding of the patient, forgetting that his daily life and personal feelings can have deep effect over the development of many musculoskeletal conditions, especially in their germinal state. In this scenario, the inclusion of humanities to the pool of multidisciplinary teams devoted to the study of gait and posture might be an important step.

In conclusion, in the age of the Anthropocene, in which humans are changing the world, which in turn is changing humans, working synergistically and multidisciplinarily on the prevention of musculoskeletal diseases seems mandatory. Looking at the human condition not only technically and medically, but promoting studies that explore the cognitive, emotional, philosophical, and also artistical expression of human motion, can be a way to turn a deep introspective gaze at our lifestyle and change it before it changes us.
